# Chronic Conditions and Sleep Problems among Adults Aged 50 years or over in Nine Countries: A Multi-Country Study

**DOI:** 10.1371/journal.pone.0114742

**Published:** 2014-12-05

**Authors:** Ai Koyanagi, Noe Garin, Beatriz Olaya, Jose Luis Ayuso-Mateos, Somnath Chatterji, Matilde Leonardi, Seppo Koskinen, Beata Tobiasz-Adamczyk, Josep Maria Haro

**Affiliations:** 1 Research and Development Unit, Parc Sanitari Sant Joan de Déu, Fundació Sant Joan de Déu, Sant Boi de Llobregat, Barcelona, Spain; 2 Department of Psychiatry, Universidad Autónoma de Madrid, Madrid, Spain; 3 Instituto de Salud Carlos III, Centro de Investigación Biomédica en Red de Salud Mental, CIBERSAM, Spain; 4 Instituto de Investigación Sanitaria Princesa (IP), Madrid, Spain; 5 Department of Health Statistics and Information Systems, World Health Organization, Geneva, Switzerland; 6 Neurology, Public Health and Disability Unit, Neurological Institute Carlo Besta IRCCS Foundation, Milan, Italy; 7 National Institute for Health and Welfare, Helsinki, Finland; 8 Department of Medical Sociology, Jagiellonian University Medical College, Krakow, Poland; University of Rochester, United States of America

## Abstract

**Background:**

Data on the association between chronic conditions or the number of chronic conditions and sleep problems in low- or middle-income countries is scarce, and global comparisons of these associations with high-income countries have not been conducted.

**Methods:**

Data on 42116 individuals 50 years and older from nationally-representative samples of the Collaborative Research on Ageing in Europe (Finland, Poland, Spain) and the World Health Organization's Study on Global Ageing and Adult Health (China, Ghana, India, Mexico, Russia, South Africa) conducted between 2011–2012 and 2007–2010 respectively were analyzed.

**Results:**

The association between nine chronic conditions (angina, arthritis, asthma, chronic lung disease, depression, diabetes, hypertension, obesity, and stroke) and self-reported severe/extreme sleep problems in the past 30 days was estimated by logistic regression with multiple variables. The age-adjusted prevalence of sleep problems ranged from 2.8% (China) to 17.0% (Poland). After adjustment for confounders, angina (OR 1.75–2.78), arthritis (OR 1.39–2.46), and depression (OR 1.75–5.12) were significantly associated with sleep problems in the majority or all of the countries. Sleep problems were also significantly associated with: asthma in Finland, Spain, and India; chronic lung disease in Poland, Spain, Ghana, and South Africa; diabetes in India; and stroke in China, Ghana, and India. A linear dose-dependent relationship between the number of chronic conditions and sleep problems was observed in all countries. Compared to no chronic conditions, the OR (95%CI) for 1,2,3, and≥4 chronic conditions was 1.41 (1.09–1.82), 2.55 (1.99–3.27), 3.22 (2.52–4.11), and 7.62 (5.88–9.87) respectively in the overall sample.

**Conclusions:**

Identifying co-existing sleep problems among patients with chronic conditions and treating them simultaneously may lead to better treatment outcome. Clinicians should be aware of the high risk for sleep problems among patients with multimorbidity. Future studies are needed to elucidate the best treatment options for comorbid sleep problems especially in developing country settings.

## Introduction

The prevalence of insomnia increases with age [1], and in Western countries, it has been reported to be as high as 50% among older adults [2]. Insomnia among the elderly is an important health issue as it has been associated with a higher risk for poorer overall health [3], lower quality of life [4], [5], and morbidity including falls [6] and institutionalization [7], and cognitive impairment [8]. Furthermore, the treatment poses a challenge especially among the elderly due to the frequent coexistence of physical and mental disorders, polypharmacy, and the side effects of drugs commonly used to treat sleep problems [9]. Indeed, up to 90% of insomnia patients have been reported to have comorbid physical and/or mental illnesses such as mood disorders, cardiovascular and respiratory diseases, hypertension, and diabetes [10], [11].

Insomnia may be the result of the chronic conditions, or may be the precursor of the chronic conditions. Alternatively, it may share the same underlying factors or be an incidental condition. Insomnia subsequent to chronic conditions has been traditionally called ‘secondary insomnia’ but due to the complexity of the association where the temporal association or causality is not always clear, the term ‘comorbid insomnia’ to denote this condition was introduced in 2005 by the National Institutes of Health (NIH) State-of-the-Science conference statement on chronic insomnia [12].

Older adults are particularly susceptible to comorbid insomnia since the change in sleep architecture associated with age, specifically lighter sleep, renders them increasingly vulnerable to sleep disruption caused by physical and mental illnesses [13]. Despite this, to date, data on comorbid sleep problems among older adults in low- or middle-income settings is scarce. This is an important research gap as the proportion of the global population 60 years and older residing in developing countries was 65% in 2010 and this figure is projected to increase to 80% by 2050 [14]. Furthermore, there is a rapid increase in chronic diseases in developing countries [15]. In addition, a progressive increase in the prevalence of sleep complaints has been observed in Brazil between 1987 and 2007 [16], and a recent large multi-country study from Asia and Africa focusing on adults aged 50 years and older reported the prevalence of severe/extreme sleep problems to be as high as 43.9% among Bangladeshi women, with prevalence figures higher than 25% observed also in Vietnam and South Africa, suggesting that there may be an under-recognized emerging global epidemic of sleep problems [17].

Thus, the aim of the current study was to assess the association between chronic conditions or the number of chronic conditions and sleep problems among older adults using nationally-representative data from nine countries which participated in the Collaborative Research on Ageing in Europe (COURAGE) survey (Finland, Poland, and Spain) and the WHO Study on global AGEing and adult health (SAGE) survey (China, Ghana, India, Mexico, Russia, and South Africa) for global comparisons. We restricted the analysis to those 50 years or older as sleep problems and chronic diseases are more prevalent in this age group [9], [18]. Understanding the epidemiology of comorbid insomnia or sleep problems and its co-existing morbidity is important to guide clinicians as insufficient sleep may also exacerbate the underlying condition and be affecting overall quality of life and wellbeing.

## Methods

We analyzed data from the COURAGE and SAGE studies. The SAGE dataset is publically available online (http://www.who.int/healthinfo/sage/en/). The details of the two surveys have been published elsewhere [19], [20]. At the time of the survey, countries in the COURAGE and SAGE surveys corresponded to high-, and low- or middle-income countries respectively according to the World Bank classification (http://data.worldbank.org/country/). Briefly, the two surveys followed the same protocol and used standardized questionnaires to collect information on health and well-being among adult populations. Nationally-representative samples were generated by multistage clustered sampling. The sample consisted of non-institutionalized adults ≥18 years of age with oversampling of those aged≥50 years. The COURAGE was conducted between 2011–2012 in Finland, Poland, and Spain, and the SAGE was conducted between 2007–2010 in China, Ghana, India, Mexico, Russia, and South Africa. The response rate ranged from 51% (Mexico) to 93% (China). All data were collected through face-to-face interviews and measurements by trained interviewers. If the interviewer judged that the participant was unable to reply to the survey questions due to limited cognitive function, a shorter questionnaire was used with a proxy respondent replying on behalf of the participant. Blood pressure was measured 2 and 3 times in the COURAGE and SAGE surveys respectively with a ≤1 minute interval using standard protocols [21]. Height and weight were measured with the use of a stadiometer and a routinely calibrated electronic weighting scale respectively. Sampling weights were generated to adjust for the population structure reported by the United Nations Statistical Division for the SAGE, and the census of the National Institute of Statistics for the COURAGE. These weights were post-stratified to weight up to the total number of individuals aged ≥18 years in each country. Ethical approval to conduct the COURAGE and SAGE surveys was obtained from local research review boards and the WHO Ethical Review Committee. Informed consent was obtained from all participants.

### Variables

Sleep problems was assessed by the single question “Overall in the last 30 days, how much of a problem did you have with sleeping, such as falling asleep, waking up frequently during the night or waking up too early in the morning?” Answer options were none, mild, moderate, severe, extreme/cannot do. Those who had severe or extreme problems were considered to have sleep problems in keeping with the approach used in a previous study from the SAGE surveys [17].

The self-reported diagnosis of eight chronic medical conditions [angina, arthritis, asthma, depression, chronic lung disease, diabetes (not associated with pregnancy), hypertension, and stroke] was based on whether the participant had ever been diagnosed to have these conditions. Body mass index (BMI) was calculated as weight in kilograms divided by height in meters squared and obesity was defined as BMI≥30 kg/m^2^. We used the combined criteria for the diagnosis of chronic conditions with the exception of diabetes, for which no information other than self-report was available, and obesity. The combined criteria referred to self-reported diagnosis and/or diagnosis based on past-12 months symptoms with the exception of hypertension which was based on blood pressure measurement, and stroke which was based on lifetime symptoms. The combined diagnosis allowed for the inclusion of those who would not have been diagnosed as having the condition based on self-report alone. Although self-report of diseases has been shown to demonstrate good agreement with medical records in developed countries [22], in settings with limited access to health care systems, under-reporting of diagnosis may be a problem. In the latter setting, patients may be less aware of their illness or may only have them detected when they are severe [23].

As for the diagnosis based on symptoms or blood pressure measurements, angina was based on the algorithms of the Rose questionnaire [24], depression was based on the algorithms of the DSM-IV for major depressive disorder, and hypertension was defined as a mean systolic blood pressure ≥140 mmHg and/or diastolic blood pressure ≥90 mmHg. The symptom-based definitions for arthritis, asthma, chronic lung disease, and stroke were based on typical clinical symptoms and were identical or similar to those used in previous publications using the same questionnaire [23], [25]–[27]. Arthritis was defined as having experienced pain, aching, stiffness, or swelling in or around the joints (like arms, hands, legs or feet) which were not related to an injury and lasted for more than a month, and having experienced stiffness lasting for less than 30 minutes in the joint in the morning after getting up from bed or after a long rest of the joint without movement which goes away after exercise or movement in the joint. Asthma was defined as having had an attack of wheezing or whistling breathing and at least one of the following: attack of wheezing after stopping exercise or some other physical activity; a feeling of tightness in the chest; experience of waking up with a feeling of tightness in the chest; attack of shortness of breath without obvious cause when not exercising or doing some physical activity. Chronic lung disease referred to having all of the following: shortness of breath at rest; coughing or wheezing for ten minutes or more at a time; coughing up sputum or phlegm for most days of the month for at least 3 months. Stroke was defined as ever having suffered from sudden onset of paralysis or weakness and loss of sensation on one side of the body for more than 24 hours. The number of chronic conditions was based on the nine aforementioned chronic conditions and was categorized as 0,1,2,3, and 4 or more.

The selection of the control variables used in the models was based on past literature [28]–[32]. These included sex, age (50–59, 60–69, 70–79,≥80 years), highest level of education completed (≤primary, secondary,≥tertiary), wealth quintiles based on country-specific income, marital status (currently married/cohabiting or not married), past-30 days alcohol consumption (no, yes), current smoking status (no, yes), and physical activity levels based on the Global Physical Activity Questionnaire (high, moderate, low).

### Statistical analysis

Participants whose information was obtained through the proxy questionnaire were excluded from the analysis as information on a large number of the variables pertaining to the current analysis was not collected. Analyses were conducted using the pooled sample including all countries and also by country. Country-wise analyses were conducted as one of our main objectives was to assess the between-country differences in terms of the association between chronic conditions and sleep problems. Chronic conditions in the main text referred to those diagnosed by the combined criteria in all analyses with the exception of data presented in Table 1 where the prevalence of chronic diseases based on both the self-reported and the combined diagnoses are shown. Analyses based solely on self-reported chronic conditions are shown only in the appendix as sensitivity analysis.

**Table 1:** Baseline characteristics of the study sample.

		COURAGE study			SAGE study					
	Overall	Finland	Poland	Spain	China	Ghana	India	Mexico	Russia	S. Africa
Age (years)										
50–59	44.9 (0.7)	33.3 (1.2)	38.3 (1.3)	32.2 (1.1)	44.9 (1.2)	39.7 (1.1)	48.6 (1.0)	48.1 (4.1)	45.2 (2.4)	49.9 (1.6)
60–69	30.1 (0.5)	35.4 (1.4)	32.9 (1.3)	28.2 (0.8)	31.9 (0.6)	27.5 (0.8)	30.9 (0.9)	25.6 (2.7)	24.6 (1.3)	30.6 (1.2)
70–79	19.0 (0.5)	20.0 (1.0)	19.9 (1.1)	29.3 (1.2)	18.6 (0.9)	23.1 (0.9)	16.0 (0.8)	17.8 (1.9)	21.8 (1.6)	14.0 (0.9)
≥80	6.0 (0.2)	11.2 (0.7)	8.8 (0.5)	10.3 (0.5)	4.6 (0.3)	9.7 (0.6)	4.5 (0.3)	8.6 (1.0)	8.4 (1.0)	5.5 (0.6)
Female	52.5 (0.5)	54.0 (1.3)	56.5 (1.1)	53.6 (1.2)	50.2 (0.7)	47.6 (1.0)	49.0 (0.6)	53.2 (3.2)	61.1 (1.5)	55.9 (1.5)
Education										
≥Tertiary	8.3 (0.6)	25.9 (2.3)	15.7 (1.1)	10.8 (0.9)	4.5 (0.7)	3.6 (0.4)	5.1 (0.6)	8.1 (1.9)	18.2 (2.3)	5.7 (0.8)
Secondary	36.1 (1.2)	56.9 (1.8)	59.3 (1.3)	25.6 (1.6)	32.5 (1.4)	21.1 (1.0)	18.8 (0.9)	12.3 (2.0)	74.2 (2.7)	22.8 (1.5)
≤Primary	55.6 (1.5)	17.2 (1.6)	25.0 (1.2)	63.6 (2.2)	63.0 (1.9)	75.3 (1.2)	76.1 (1.3)	79.6 (2.9)	7.5 (0.9)	71.4 (1.7)
Married/cohabiting	74.1 (0.8)	64.7 (1.8)	68.1 (1.2)	62.4 (1.1)	85.0 (0.6)	59.3 (1.1)	76.9 (0.8)	73.0 (3.2)	58.3 (2.9)	55.9 (1.5)
Current drinker	22.6 (0.7)	63.5 (1.8)	46.8 (1.4)	46.6 (1.3)	22.9 (1.0)	30.6 (1.3)	6.8 (0.5)	24.8 (3.8)	32.7 (2.5)	13.7 (1.1)
Current smoker	33.2 (0.8)	17.4 (1.1)	26.1 (1.3)	19.8 (0.9)	29.3 (0.9)	10.7 (0.7)	50.0 (1.2)	20.3 (3.0)	21.3 (2.0)	23.8 (1.3)
Physical activity										
High	47.8 (0.9)	41.7 (2.1)	48.6 (1.5)	30.6 (1.6)	43.8 (1.6)	61.5 (1.5)	52.3 (1.6)	38.8 (4.7)	57.3 (2.7)	27.4 (1.7)
Moderate	23.4 (0.6)	31.5 (1.4)	19.6 (1.1)	37.8 (1.4)	27.0 (1.1)	12.5 (0.8)	22.8 (1.0)	21.7 (3.2)	15.6 (1.3)	11.9 (0.8)
Low	28.8 (0.7)	26.8 (1.7)	31.7 (1.5)	31.6 (1.2)	29.2 (1.3)	26.1 (1.4)	25.0 (1.1)	39.5 (3.9)	27.1 (2.2)	60.7 (1.8)

Abbreviations: COURAGE Collaborative Research on Ageing in Europe; SAGE WHO Study on global AGEing and adult health; S. Africa South Africa.

Data are % (SE) unless otherwise stated.

The crude prevalence of baseline characteristics of the study sample was calculated (Table 1). The age-adjusted prevalence of sleep problems and chronic conditions was calculated using the United Nation population pyramids for the year 2010 (http://esa.un.org/wpp/Excel-Data/population.htm) as the standard population (Table 2). The difference in the prevalence of sleep problems by the presence or absence of the individual chronic conditions was tested by chi-squared tests (Table 3). Logistic regression analyses with multiple variables were done to assess the association between the chronic health conditions or the number of chronic health conditions (independent variable) and sleep problems (dependent variable). In the analysis assessing the association between the individual chronic health conditions and sleep problems, the models were mutually adjusted for the nine chronic conditions in addition to age, sex, education, wealth, marital status, alcohol consumption, smoking, and physical activity (Table 4). The nine chronic conditions were included simultaneously in the models as these conditions tend to co-occur [25], and have all been associated with sleep problems [10], [33]–[46]. In the analysis on the association between the number of chronic health conditions and sleep problems, the models were adjusted for age, sex, education, wealth, marital status, alcohol consumption, smoking, and physical activity (Table 5). A test for trend was conducted by including the number of chronic conditions in the model as a continuous variable. Adjustment for country was also done for regression models that pooled data of all countries by including countries as dummy variables in the models. The sample weighting and the complex study design were taken into account in all analyses to generate nationally-representative estimates using the Stata *svy* command. We analyzed data with Stata version 12.1 (Stata Corp LP, College Station, Texas). The level of statistical significance was set at P<0.05.

**Table 2:** Age-adjusted prevalence of severe/extreme sleep problems and chronic conditions among adults aged 50 years or over.

		COURAGE study			SAGE study					
	Overall	Finland	Poland	Spain	China	Ghana	India	Mexico	Russia	S. Africa
**Severe/extreme sleep problems**	9.2 (0.4)	9.6 (0.8)	17.0 (0.9)	8.1 (0.8)	2.8 (0.2)	7.1 (0.5)	15.0 (0.9)	5.8 (1.2)	9.0 (1.0)	9.6 (0.7)
**Angina**										
Self-reported	11.3 (0.7)	9.3 (0.8)	13.0 (0.9)	5.3 (0.6)	7.9 (0.4)	3.4 (0.3)	5.5 (0.7)	2.8 (0.9)	31.0 (2.3)	5.2 (0.6)
Self-reported and/or symptoms	16.8 (0.8)	9.9 (0.7)	18.3 (1.0)	6.7 (0.7)	9.3 (0.4)	12.4 (0.7)	17.2 (1.3)	6.9 (1.2)	36.0 (2.3)	8.9 (0.8)
**Arthritis**										
Self-reported	23.2 (0.6)	43.6 (1.2)	30.3 (1.3)	25.1 (1.2)	21.8 (0.6)	13.0 (1.0)	18.4 (0.9)	9.0 (1.4)	29.4 (2.3)	24.7 (1.4)
Self-reported and/or symptoms	30.1 (0.7)	47.3 (1.3)	34.2 (1.4)	29.1 (1.3)	26.5 (0.7)	25.3 (1.2)	28.0 (0.9)	14.7 (1.6)	37.5 (2.6)	30.6 (1.5)
**Asthma**										
Self-reported	4.5 (0.3)	10.9 (1.0)	5.6 (0.5)	6.9 (0.4)	2.0 (0.1)	3.1 (0.3)	7.3 (0.6)	1.9 (0.3)	2.7 (0.5)	4.8 (0.6)
Self-reported and/or symptoms	8.1 (0.4)	12.9 (1.0)	9.4 (0.7)	9.7 (0.6)	4.3 (0.3)	4.8 (0.4)	12.6 (0.8)	4.9 (0.8)	6.6 (0.9)	7.7 (0.7)
**Chronic lung disease**										
Self-reported	7.6 (0.4)	3.5 (0.6)	7.8 (0.6)	6.7 (0.6)	8.0 (0.3)	0.5 (0.1)	4.5 (0.7)	3.7 (0.6)	14.7 (1.4)	2.9 (0.5)
Self-reported and/or symptoms	9.1 (0.4)	4.3 (0.7)	8.7 (0.6)	7.6 (0.7)	8.3 (0.3)	1.2 (0.2)	8.1 (0.8)	4.6 (0.7)	15.1 (1.4)	4.2 (0.6)
**Depression**										
Self-reported	4.3 (0.3)	14.8 (0.9)	11.1 (0.9)	22.6 (1.1)	0.3 (0.1)	1.7 (0.3)	4.0 (0.5)	13.5 (2.6)	3.5 (0.7)	2.8 (0.4)
Self-reported and/or symptoms	9.4 (0.5)	16.0 (0.8)	13.7 (1.0)	26.4 (1.2)	1.3 (0.1)	7.6 (0.7)	16.0 (0.9)	16.7 (2.7)	5.6 (0.8)	4.7 (0.6)
**Diabetes**										
Self-reported	7.8 (0.3)	11.6 (0.9)	12.7 (0.8)	14.4 (0.9)	6.5 (0.3)	3.8 (0.4)	6.9 (0.6)	18.2 (2.0)	6.9 (0.9)	9.4 (0.8)
**Hypertension**										
Self-reported	30.3 (0.8)	38.0 (1.3)	50.7 (1.3)	36.3 (1.1)	26.7 (0.5)	13.9 (0.7)	17.0 (0.8)	31.3 (2.4)	51.8 (2.6)	30.9 (1.6)
Self-reported and/or measurement	56.1 (0.8)	63.8 (1.5)	65.8 (1.3)	58.8 (1.1)	60.5 (0.8)	59.5 (1.2)	37.5 (1.0)	62.1 (2.7)	71.1 (2.2)	78.6 (1.1)
**Obesity**										
Measurement	14.4 (0.8)	26.0 (1.3)	34.7 (1.4)	31.0 (1.1)	5.8 (0.4)	10.4 (0.7)	2.5 (0.3)	28.8 (3.1)	35.0 (3.0)	46.3 (1.8)
**Stroke**										
Self-reported	3.3 (0.2)	4.2 (0.6)	4.5 (0.5)	3.6 (0.4)	3.1 (0.2)	2.6 (0.3)	2.0 (0.2)	4.5 (0.9)	4.5 (0.6)	4.1 (0.6)
Self-reported and/or symptoms	4.4 (0.3)	4.5 (0.7)	5.4 (0.6)	4.3 (0.5)	3.7 (0.2)	3.5 (0.3)	3.9 (0.4)	5.8 (0.9)	6.2 (1.2)	5.0 (0.7)

Abbreviations: COURAGE Collaborative Research on Ageing in Europe; SAGE WHO Study on global AGEing and adult health; S. Africa South Africa.

Data are % (SE) unless otherwise stated.

**Table 3:** Prevalence of severe/extreme sleep problems by presence of chronic condition and country among adults aged 50 years or over.

			COURAGE study								SAGE study									
	Overall		Finland		Poland		Spain		China		Ghana		India		Mexico		Russia		S. Africa	
Chronic condition	Yes	No	Yes	No	Yes	No	Yes	No	Yes	No	Yes	No	Yes	No	Yes	No	Yes	No	Yes	No
Angina^a,b,c,d,e,f,g,h,i,j^	19.7	7.1	22.3	7.9	36.5	13.3	19.8	8.0	6.4	2.4	13.2	6.6	27.5	11.9	12.8	5.1	16.8	5.4	21.4	8.2
	(1.3)	(0.3)	(3.5)	(0.7)	(2.7)	(1.0)	(2.8)	(0.8)	(0.8)	(0.2)	(1.8)	(0.5)	(2.2)	(0.7)	(4.0)	(1.3)	(2.4)	(1.0)	(3.5)	(0.7)
Arthritis^a,b,c,d,e,f,g,i,j^	14.7	6.9	14.4	5.0	26.5	13.0	16.5	5.3	4.3	2.2	11.3	6.0	21.3	11.9	7.4	5.3	16.1	5.7	14.3	7.3
	(0.8)	(0.4)	(1.2)	(0.7)	(1.9)	(1.1)	(1.5)	(0.7)	(0.5)	(0.2)	(1.2)	(0.6)	(1.7)	(0.8)	(1.6)	(1.4)	(2.4)	(0.8)	(1.6)	(0.8)
Asthma^a,b,c,d,e,f,g,i,j^	22.5	8.1	22.1	7.9	33.9	16.0	21.9	7.5	7.6	2.5	11.7	7.2	27.5	12.7	7.4	5.6	19.8	9.0	20.4	8.5
	(1.8)	(0.4)	(3.3)	(0.6)	(3.8)	(1.0)	(2.3)	(0.7)	(2.1)	(0.2)	(2.3)	(0.6)	(3.3)	(0.7)	(2.4)	(1.3)	(3.8)	(1.1)	(3.9)	(0.7)
Chronic lung disease^a,b,c,d,e,f,g,h,i,j^	17.5	8.4	17.8	9.3	36.4	15.9	24.4	7.5	5.2	2.5	25.6	7.2	23.4	13.7	11.4	5.4	18.7	8.1	29.7	8.5
	(1.2)	(0.4)	(4.8)	(0.7)	(3.8)	(1.0)	(2.7)	(0.7)	(0.9)	(0.2)	(6.5)	(0.5)	(2.6)	(0.8)	(2.9)	(1.3)	(2.5)	(1.1)	(6.2)	(0.7)
Diabetes^a,b,c,d,g,h,i^	14.0	8.9	14.2	9.0	23.3	16.9	12.6	8.2	3.6	2.7	8.8	7.4	21.2	14.0	11.6	4.4	15.8	9.2	12.1	9.1
	(1.1)	(0.4)	(2.5)	(0.7)	(2.5)	(1.1)	(1.5)	(0.8)	(0.9)	(0.2)	(2.3)	(0.6)	(3.0)	(0.9)	(5.0)	(1.0)	(2.6)	(1.1)	(2.7)	(0.8)
Depression^a,b,c,d,e,f,g,h,i,j^	26.8	7.4	21.3	7.4	35.4	15.0	20.6	4.8	17.7	2.5	15.3	6.7	27.9	12.0	13.6	4.0	31.3	8.4	31.4	8.3
	(1.3)	(0.4)	(3.1)	(0.7)	(3.1)	(1.0)	(2.0)	(0.5)	(3.5)	(0.2)	(2.7)	(0.6)	(2.1)	(0.9)	(5.2)	(1.2)	(4.1)	(1.0)	(6.3)	(0.7)
Hypertension^a,c,g,h,i^	10.0	8.3	10.6	7.6	19.8	13.7	9.9	7.4	2.7	2.8	7.8	6.9	17.9	12.5	7.2	3.1	11.6	5.0	9.2	9.7
	(0.5)	(0.5)	(1.0)	(1.1)	(1.2)	(1.5)	(1.0)	(1.0)	(0.2)	(0.3)	(0.7)	(0.7)	(1.2)	(1.0)	(1.8)	(1.0)	(1.4)	(1.1)	(0.8)	(1.5)
Obesity^ a,c,d^	11.2	8.6	10.2	9.0	21.6	14.8	11.7	6.9	3.2	2.7	8.1	6.9	11.2	14.2	4.1	6.3	10.5	8.2	9.3	9.3
	(0.9)	(0.4)	(1.6)	(0.8)	(2.0)	(1.1)	(1.4)	(0.7)	(0.7)	(0.2)	(1.6)	(0.5)	(2.9)	(0.8)	(1.0)	(1.9)	(1.9)	(1.2)	(1.2)	(1.0)
Stroke^a,b,c,e,f,g,h,i,j^	19.8	8.8	19.2	9.1	27.5	17.2	13.8	8.7	7.2	2.6	20.8	6.9	31.3	13.9	12.0	5.3	21.6	8.9	18.4	8.9
	(2.1)	(0.4)	(4.4)	(0.7)	(4.6)	(1.0)	(4.2)	(0.8)	(1.7)	(0.2)	(3.7)	(0.5)	(4.4)	(0.9)	(3.4)	(1.3)	(6.1)	(1.1)	(5.2)	(0.7)

Abbreviation: COURAGE Collaborative Research on Ageing in Europe; SAGE WHO Study on global AGEing and adult health; S. Africa South Africa

Data are % (SE). % is the percentage of individuals with sleep problems by the presence (Yes) or absence (No) of that chronic condition.

Difference between individuals with and without that chronic condition is statistically significant (P<0.05) in ^a^Overall sample, ^b^Finland, ^c^Poland, ^d^Spain,^ e^China, ^f^Ghana, ^g^India, ^h^Mexico, ^i^Russia, and ^j^South Africa.

**Table 4:** Association between chronic conditions (independent variable) and severe/extreme sleep problems (dependent variable) among adults aged 50 years or over estimated by logistic regression with multiple variables.

		COURAGE study			SAGE study					
	Overall	Finland	Poland	Spain	China	Ghana	India	Mexico	Russia	S. Africa
Angina	2.02***	2.78***	2.36***	1.78*	1.75**	1.92***	1.91***	1.84	2.49***	2.20**
	(1.70–2.40)	(1.55–4.96)	(1.60–3.47)	(1.12–2.83)	(1.22–2.51)	(1.35–2.72)	(1.44–2.52)	(0.72–4.70)	(1.52–4.07)	(1.25–3.88)
Arthritis	1.54***	2.46***	1.49*	1.70*	1.39*	1.57**	1.43**	0.96	1.97**	1.28
	(1.33–1.78)	(1.64–3.68)	(1.08–2.04)	(1.08–2.65)	(1.08–1.78)	(1.12–2.20)	(1.13–1.80)	(0.50–1.84)	(1.27–3.06)	(0.79–2.07)
Asthma	1.68***	2.31**	1.15	1.56*	1.75	1.26	2.13***	0.62	1.09	1.68
	(1.28–2.22)	(1.40–3.82)	(0.73–1.80)	(1.05–2.34)	(0.89–3.44)	(0.73–2.20)	(1.41–3.20)	(0.24–1.63)	(0.59–2.01)	(0.99–2.86)
Chronic lung disease	1.32	0.98	2.10***	2.21***	1.40	3.74***	0.81	1.41	1.82	3.47***
	(1.00–1.75)	(0.40–2.43)	(1.41–3.12)	(1.51–3.23)	(0.92–2.13)	(1.72–8.12)	(0.49–1.34)	(0.60–3.31)	(0.98–3.40)	(1.84–6.52)
Depression	2.36***	2.62***	2.22***	4.03***	4.63***	1.75*	1.94***	5.12***	3.15***	3.44*
	(1.95–2.86)	(1.60–4.28)	(1.58–3.12)	(2.91–5.59)	(2.76–7.77)	(1.02–3.01)	(1.45–2.58)	(2.42–10.83)	(1.97–5.06)	(1.27–9.30)
Diabetes	1.30*	1.19	0.95	1.09	1.11	1.09	1.82**	2.02	1.18	1.27
	(1.06–1.60)	(0.66–2.13)	(0.64–1.40)	(0.71–1.68)	(0.70–1.76)	(0.58–2.04)	(1.26–2.61)	(0.92–4.42)	(0.71–1.96)	(0.64–2.52)
Hypertension	1.12	1.50	1.20	1.10	0.77	0.97	1.24	1.38	1.03	0.96
	(0.95–1.32)	(0.88–2.55)	(0.84–1.71)	(0.78–1.56)	(0.56–1.06)	(0.72–1.31)	(0.98–1.56)	(0.68–2.80)	(0.64–1.66)	(0.60–1.52)
Obesity	1.05	0.68	1.21	1.18	1.08	1.38	0.67	0.56	1.11	0.92
	(0.86–1.27)	(0.41–1.15)	(0.84–1.73)	(0.82–1.71)	(0.66–1.78)	(0.90–2.13)	(0.38–1.21)	(0.28–1.14)	(0.73–1.70)	(0.59–1.41)
Stroke	1.81***	1.72	1.26	0.57	2.11*	2.14*	2.35***	1.68	1.66	1.49
	(1.34–2.44)	(0.86–3.42)	(0.68–2.35)	(0.27–1.21)	(1.14–3.91)	(1.17–3.91)	(1.43–3.84)	(0.71–3.99)	(0.94–2.94)	(0.47–4.74)

Abbreviation: COURAGE Collaborative Research on Ageing in Europe; SAGE WHO Study on global AGEing and adult health; S. Africa South Africa.

Data are Odds Ratio (95% Confidence Intervals).

All models are mutually adjusted for all chronic conditions in the model and age, sex, education, wealth, marital status, alcohol consumption, smoking, and physical activity. The model using the overall sample is also adjusted for county.

*p<0.05,

**p<0.01,

***p<0.001.

**Table 5:** Association between number of chronic conditions (independent variable) and severe/extreme sleep problems (dependent variable) estimated by logistic regression with multiple variables.

No. of chronic conditions		COURAGE study			SAGE study					
	Overall	Finland	Poland	Spain	China	Ghana	India	Mexico	Russia	S. Africa
0 (Reference)	1.00	1.00	1.00	1.00	1.00	1.00	1.00	1.00	1.00	1.00
1	1.41*	1.60	1.97*	3.93**	0.84	1.13	1.41	0.80	3.18	1.37
	(1.09–1.82)	(0.47–5.46)	(1.02–3.79)	(1.68–9.21)	(0.53–1.33)	(0.72–1.79)	(0.99–2.01)	(0.21–3.03)	(0.91–11.15)	(0.55–3.40)
2	2.55***	3.03	2.65**	4.94***	1.60*	2.17**	2.91***	2.00	5.01*	1.79
	(1.99–3.27)	(0.88–10.47)	(1.35–5.20)	(2.01–12.17)	(1.00–2.55)	(1.36–3.47)	(2.09–4.07)	(0.61–6.56)	(1.30–19.26)	(0.69–4.67)
3	3.22***	4.55*	3.92***	9.80***	1.22	2.93***	3.27***	2.42	8.55**	2.99*
	(2.52–4.11)	(1.42–14.55)	(1.98–7.75)	(4.19–22.94)	(0.72–2.07)	(1.74–4.94)	(2.35–4.55)	(0.70–8.45)	(2.33–31.38)	(1.05–8.51)
4+	7.62***	10.91***	8.50***	17.95***	4.53***	4.85***	7.71***	3.70	19.20***	7.10***
	(5.88–9.87)	(3.14–37.91)	(4.34–16.65)	(7.72–41.75)	(2.57–7.97)	(2.52–9.32)	(5.25–11.32)	(0.92–14.93)	(5.64–65.37)	(2.63-19.13)

Abbreviation: COURAGE Collaborative Research on Ageing in Europe; SAGE WHO Study on global AGEing and adult health; S. Africa South Africa.

Data are Odds Ratio (95% Confidence Intervals).

Trend test was significant for all regression analyses (p≤0.002).

All models are mutually adjusted for age, sex, education, wealth, marital status, alcohol consumption, smoking, and physical activity. The model using the overall sample is also adjusted for county.

*p<0.05,

**p<0.01,

***p<0.001.

## Results

The analytical sample size, after excluding those whose information was obtained by the proxy questionnaire and individuals aged<50 years, was 42116 (China 13175, Finland 1452, Ghana 4305, India 6560, Mexico 2313, Poland 2910, Russia 3938, South Africa 3838, Spain 3625).

The baseline characteristics of the study sample are illustrated in **Table 1**. The median age ranged from 60 years (India, Mexico, and South Africa) to 65 years (Spain), and the proportion of females was particularly high in Russia. The age-adjusted prevalence of severe/extreme sleep problems and chronic conditions are demonstrated in **Table 2**. The overall prevalence of sleep problems was 9.2% and ranged from 2.8% (China) to 17.0% (Poland) with the highest prevalence observed in India (15.0%) among the SAGE countries. A large between-country difference in terms of the prevalence of some chronic conditions was observed. For example, the prevalence of self-reported angina ranged from 2.8% (Mexico) to 31.0% (Russia), and the corresponding figures for self-reported depression was 0.3% (China) to 22.6% (Spain). The difference between the prevalence of chronic conditions based on self-report only and the combined criteria was particularly pronounced in the SAGE countries. For instance, in Ghana, as compared to self-report, the prevalence based on the combined diagnosis lead to an increase from 3.4% to 12.4%, 1.7% to 7.6%, and 13.9% to 59.5% for angina, depression, and hypertension respectively. The prevalence of sleep problems by the presence of chronic conditions (univariate analysis) is illustrated in **Table 3**. In the univariate analysis, all conditions were significantly associated with higher likelihoods for sleep problems in the overall sample. Angina, chronic lung disease, and depression were significantly associated with higher prevalence of sleep problems in all individual countries as well. In the majority of countries, a significantly greater likelihood for sleep problems was observed for all the other chronic conditions as well although this association for hypertension and obesity were observed in fewer countries. The associations between sleep problems and chronic conditions estimated by logistic regression with multiple variables are illustrated in **Table 4** (results for all covariates in the models are available in Table S1). In the overall sample, all chronic conditions except chronic lung disease, hypertension, and obesity were significantly associated with sleep problems. Angina was associated with significant 1.75–2.78 times higher odds for sleep problems in all countries except Mexico. Similarly, arthritis was significantly associated with 1.39–2.46 times higher odds for sleep problems in all countries with the exception of Mexico and South Africa. The likelihood for sleep problems was significantly higher among asthmatics in Finland, Spain, and India. As for chronic lung disease, significantly higher odds for sleep problems were observed in Poland, Spain, Ghana, and South Africa. Depression was linked with higher odds for sleep problems in all countries. Diabetes was associated with a significant 1.82 times higher odds for sleep problems only in India. Hypertension and obesity were not associated with sleep problems in any of the countries. A significant association between stroke and sleep problems was observed in China, Ghana, and India. The association between the number of chronic conditions and sleep problems is shown in **Table 5** (results for all covariates in the models are available in Table S2 of the Appendix). A significant linear dose-dependent relationship between the number of chronic conditions and sleep problems was observed in all samples. Finally, the results of the sensitivity analyses based solely on self-reported diagnoses were generally similar to the main analysis with changes in statistical significance occurring more commonly in the SAGE countries than the COURAGE countries (Appendix Table S3, S4, S5). In particular, self-reported hypertension was associated with sleep problems in Finland, India, and Mexico despite the fact that hypertension based on self-report and/or blood pressure measurement was not associated with sleep problems in any of the countries.

## Discussion

Our study has shown that sleep problems among older adults were common in countries such as Poland (17.0%), India (15.0%), and Russia (9.0%), and that many common chronic conditions are associated with sleep problems even after adjustment for confounders. No distinct patterns regarding the association between sleep problems and individual chronic conditions were observed by region or the level of economic development of the country. The strength of our study is the large sample size and the use of nationally-representative datasets from diverse settings. Also, our diagnostic definition incorporating symptoms or blood pressure measurement is likely to have reduced under-diagnosis especially in resource-limited settings. We also adjusted for depression which many previous studies failed to do despite its strong association with sleep and physical illness [47]. To the best of our knowledge, this is the first multi-continent study to examine the association between chronic conditions and sleep problems.

Several limitations deserve mentioning before discussing the results. There are no standard definitions for insomnia or sleep problems in epidemiological research [48]. Although we used a single-item question to define sleep problems, the question encompassed the three dimensions of insomnia (i.e. problems falling asleep, waking up frequently during the night, and waking up too early in the morning) [49]. Also, the use of severe or extreme categories to define sleep problems is likely to have improved the specificity. However, we were unable to conduct detailed analyses on the severity, type, or chronicity of the sleep problem which would have lead to better understanding of the association between sleep problems and chronic conditions. Next, we attempted to reduce under-diagnosis of chronic conditions by the use of an algorithm based on symptoms but the use of non-validated instruments for arthritis, asthma, chronic lung diseases, and stroke may have lead to higher sensitivity at the expense of lower specificity. However, in order to minimize misclassification, we used algorithms based on current clinical guidelines and reference publications [25]. Furthermore, due to the high potential for under-diagnosis especially in resource-limited settings, as evidenced by a higher rate of discrepancy between the two diagnostic definitions especially in the SAGE countries, we believe that this procedure was necessary and justifiable to address our study question. Next, due to the difficulty in differentiating chronic lung diseases such as COPD and asthma because of the overlapping symptoms especially among older populations [50], varying degrees of misclassification depending on the setting is likely to have occurred. Also, we did not have information on caffeine intake or conditions such as anxiety disorder, sleep apnea, fibromyalgia, migraine, restless leg syndrome, and neurological disorders known to be associated with sleep problems [10], [48], [51]. Thus, their independent and confounding effects remain unknown. In addition, some country-wise analyses may have lacked statistical power due to small sample size. Moreover, the COURAGE and the SAGE surveys were conducted in different years ranging from 2007 to 2012. Thus, the data may not reflect the current situation in some countries. Finally, because this was a cross-sectional study, causality cannot be inferred.

Data from high-income countries have reported associations between sleep disturbance and angina [33], arthritis [34]–[37], asthma [36]–[38], chronic lung disease [37], [39], diabetes [37], [40]–[42], depression [10], hypertension [37], [43], obesity [44], and stroke [45], although the results for hypertension [36], [43], [46] are less consistent. One of the only studies from developing countries on comorbid insomnia conducted in Nigeria reported a significant association between insomnia and arthritis, heart disease, stroke, high blood pressure, asthma, but not diabetes [52]. In our study, angina, arthritis, chronic lung disease, and depression were significantly associated with sleep problems in the majority or all of the countries studied, and significant associations between other chronic conditions were also observed in fewer countries with the exception of hypertension and obesity.

The associations between sleep problems and the chronic conditions may be explained by the co-existence of sleep-disordered breathing in conditions such as chronic lung disease, diabetes, and stroke [53], [54], or symptoms of the chronic conditions per se (e.g. nocturnal symptoms in asthma [38], COPD [54], and angina [55], and nocturia in diabetes [56]). Furthermore, recent studies have pointed to the potential role of insomnia in the future occurrence of metabolic and cardiovascular diseases [43], [57], [58]. The association between arthritis and sleep problems may be mediated by pain [34], [35] and physical limitation [35]. Depression is the condition most likely to co-exist with chronic insomnia [10], and sleep problem is one of the core symptoms of depression, which has also been linked to the subsequent onset of many psychiatric disorders including depression [59]. Finally, these associations may also be explained by psychological distress and anxiety associated with these conditions [60], [61].

Although the reason for between-country differences in the association between chronic conditions and sleep problems is unclear, one may speculate several reasons. First of all, the question on sleep may have been interpreted differently between countries. Secondly, the accuracy of the diagnosis of chronic medical condition or its severity may have differed depending on the country. Furthermore, non-pharmacologic or pharmacologic treatment [62] for insomnia may have been more common in some settings. Finally, a variety of drugs to treat chronic conditions, such as SSRIs and beta-blockers, may induce insomnia [10] and these drugs may have been more commonly prescribed in some areas.

Previously, treating the comorbid condition was believed to lead to the improvement in sleep conditions. However, evidence is emerging that this might not be the case, and that better treatment outcome may be expected if insomnia is treated separately, possibly even for the underlying chronic condition [10]. Although most previous clinical trials demonstrating the added benefits of treating insomnia in conjunction to the co-existing morbidity are on mental disorders [11], or arthritis [63], [64], sleep deprivation among those with COPD has been reported to lead to a small fall in forced expiratory volume and forced vital capacity [65], and studies have also shown that short sleep may impair glucose tolerance among individuals with and without diabetes [66]. Furthermore, sleep problems may affect immunity, inflammation or cause distress, which may contribute to severity of disease [37].

There are currently no treatment algorithms specifically for comorbid insomnia, but several treatment options for comorbid insomnia have been proposed and include pharmacologic, and non-pharmacologic treatments such as cognitive behavioral therapy, sleep hygiene, and relaxation training [11]. In resource-limited settings, many of the hypnotics used in developed country settings may not be available [62], and cost-effectiveness is a more crucial issue.

In conclusion, this study has shown that comorbid sleep problems affect individuals in developing and developed countries alike. Longitudinal studies especially in low- or middle-income countries, where only limited data exist [67], are warranted to understand the cause-and-effect relationships between chronic conditions and sleep problems. Medical personnel should consider the possibility of co-existing mental or physical conditions for patients presenting with symptoms of insomnia, and for those patients with a chronic condition, identifying co-existing sleep problems and treating them simultaneously may lead to better treatment outcome. Also, clinicians should be aware of the particularly high risk of sleep problems among those with multimorbidity. Future studies are needed to elucidate the best treatment options for comorbid sleep problems especially in resource-limited settings.

## Supporting Information

Table S1 **Association between chronic conditions (independent variable) or other covariates and severe/extreme sleep problems (dependent variable) among adults aged 50 years or over estimated by logistic regression with multiple variables.**
(DOCX)

Table S2 **Association between number of chronic conditions (independent variable) or other covariates and severe/extreme sleep problems.**
(DOCX)

Table S3 **Prevalence of severe/extreme sleep problems by presence of chronic condition and country among adults aged 50 years or over (self-reported diagnosis).**
(DOCX)

Table S4 **Association between chronic conditions (independent variable) and severe/extreme sleep problems (dependent variable) among adults aged 50 years or over estimated by logistic regression with multiple variables (self-reported diagnosis).**
(DOCX)

Table S5 **Association between number of chronic conditions (independent variable) and severe/extreme sleep problems (dependent variable) estimated by logistic regression with multiple variables (self-reported diagnosis).**
(DOCX)
