# Correction: Chronic Conditions and Sleep Problems among Adults Aged 50 years or over in Nine Countries: A Multi-Country Study

**DOI:** 10.1371/journal.pone.0138261

**Published:** 2015-09-17

**Authors:** Ai Koyanagi, Noe Garin, Beatriz Olaya, Jose Luis Ayuso-Mateos, Somnath Chatterji, Matilde Leonardi, Seppo Koskinen, Beata Tobiasz-Adamczyk, Josep Maria Haro

In the Funding section, the grant information pertaining to Ai Koyanagi’s work was listed incorrectly.

The correct information is: Ai Koyanagi's work was supported by the Miguel Servet contract financed by the CP13 / 00150 project, integrated into the National R + D + I and funded by the ISCIII—General Branch Evaluation and Promotion of Health Research—and the European Regional Development Fund (ERDF-FEDER).

Please view the complete, correct Funding section here:

Ai Koyanagi's work was supported by the Miguel Servet contract financed by the CP13 / 00150 project, integrated into the National R + D + I and funded by the ISCIII—General Branch Evaluation and Promotion of Health Research—and the European Regional Development Fund (ERDF-FEDER). WHO's Study on Global AGEing and Adult Health is supported by the United States National Institute on Aging's Division of Behavioral and Social Research through Interagency Agreements (OGHA 04034785; YA1323-08-CN-0020; Y1-AG- 1005–01) and through a research grant (R01-AG034479) and the World Health Organization's Department of Health Statistics and Information Systems. The research leading to these results has received funding from the European Community's Seventh Framework Programme (FP7/2007–2013) under grant agreement number 223071 (COURAGE in Europe), from the Instituto de Salud Carlos III-FIS research grants number PS09/00295 and PS09/01845, and from the Spanish Ministry of Science and Innovation ACI-Promociona (ACI2009–1010). The study was also supported by the Centro de Investigación Biomédica en Red de Salud Mental (CIBERSAM), Instituto de Salud Carlos III. The views expressed in this paper are those of the author(s) and do not necessarily represent the views or policies of the World Health Organization. The funders had no role in study design, data collection and analysis, decision to publish, or preparation of the manuscript.
