# Supplementary material for: Chronic Conditions and Sleep Problems among Adults Aged 50 years or over in Nine Countries: A Multi-Country Study
Source: PLoS One. 2014 Dec 5;9(12):e114742. doi: 10.1371/journal.pone.0114742 (PMC4257709; doi:10.1371/journal.pone.0114742)
Supplement: Table S1 — Association between chronic conditions (independent variable) or other covariates and severe/extreme sleep problems (dependent variable) among adults aged 50 years or over estimated by logistic regression with multiple variables. (DOCX) [file pone.0114742.s001.docx]

| **Table S1** Association between chronic conditions (independent variable) or other covariates and severe/extreme sleep problems (dependent variable) | | | | | | | | | | | |
| --- | --- | --- | --- | --- | --- | --- | --- | --- | --- | --- | --- |
| among adults aged 50 years or over estimated by logistic regression with multiple variables | | | | | | | | | | | |
|  |  | COURAGE study | | |  | SAGE study | | | | | |
|  | Overall | Finland | Poland | Spain |  | China | Ghana | India | Mexico | Russia | S. Africa |
| Age (years) |  |  |  |  |  |  |  |  |  |  |  |
| 50-59 | 1.00 | 1.00 | 1.00 | 1.00 |  | 1.00 | 1.00 | 1.00 | 1.00 | 1.00 | 1.00 |
| 60-69 | 1.29** | 0.50* | 1.15 | 0.92 |  | 1.12 | 1.31 | 1.30* | 8.67*** | 2.30** | 0.79 |
|  | (1.10-1.52) | (0.27-0.91) | (0.79-1.68) | (0.62-1.36) |  | (0.78-1.61) | (0.89-1.92) | (1.01-1.66) | (2.45-30.64) | (1.26-4.22) | (0.47-1.33) |
| 70-79 | 1.43*** | 0.51 | 1.60* | 1.50 |  | 1.09 | 1.58* | 1.38* | 4.83* | 2.52** | 1.22 |
|  | (1.18-1.74) | (0.24-1.09) | (1.02-2.53) | (0.96-2.37) |  | (0.74-1.61) | (1.03-2.40) | (1.01-1.89) | (1.38-16.85) | (1.35-4.70) | (0.60-2.49) |
| >80 | 2.02*** | 1.14 | 1.47 | 0.95 |  | 1.59 | 2.04* | 2.76*** | 8.98*** | 3.53*** | 1.56 |
|  | (1.51-2.70) | (0.55-2.33) | (0.87-2.48) | (0.46-1.95) |  | (0.91-2.77) | (1.16-3.56) | (1.56-4.87) | (2.46-32.79) | (1.76-7.08) | (0.67-3.63) |
| Sex |  |  |  |  |  |  |  |  |  |  |  |
| Male | 1.00 | 1.00 | 1.00 | 1.00 |  | 1.00 | 1.00 | 1.00 | 1.00 | 1.00 | 1.00 |
| Female | 1.81*** | 1.33 | 1.26 | 1.76* |  | 1.60* | 1.09 | 1.87*** | 2.04 | 2.14*** | 2.37*** |
|  | (1.55-2.12) | (0.84-2.11) | (0.91-1.73) | (1.13-2.72) |  | (1.09-2.35) | (0.77-1.54) | (1.45-2.42) | (0.98-4.26) | (1.44-3.20) | (1.51-3.72) |
| Education |  |  |  |  |  |  |  |  |  |  |  |
| >Tertiary | 1.00 | 1.00 | 1.00 | 1.00 |  | 1.00 | 1.00 | 1.00 | 1.00 | 1.00 | 1.00 |
| Secondary | 0.93 | 1.50 | 1.25 | 1.73 |  | 0.80 | 1.27 | 1.16 | 9.96** | 0.66 | 0.70 |
|  | (0.69-1.25) | (0.84-2.68) | (0.79-2.00) | (0.82-3.65) |  | (0.38-1.68) | (0.54-2.97) | (0.55-2.43) | (1.86-53.24) | (0.38-1.14) | (0.12-3.91) |
| <Primary | 1.41* | 1.14 | 1.31 | 2.13 |  | 1.23 | 1.35 | 1.92 | 5.78** | 1.29 | 1.21 |
|  | (1.05-1.89) | (0.49-2.64) | (0.81-2.10) | (0.98-4.64) |  | (0.55-2.73) | (0.52-3.52) | (0.91-4.08) | (1.65-20.22) | (0.73-2.28) | (0.23-6.32) |
| Wealth |  |  |  |  |  |  |  |  |  |  |  |
| Poorest | 1.17 | 1.32 | 1.53 | 1.23 |  | 1.02 | 1.21 | 1.17 | 2.33 | 1.35 | 1.18 |
|  | (0.92-1.48) | (0.64-2.69) | (0.93-2.50) | (0.71-2.14) |  | (0.66-1.60) | (0.77-1.89) | (0.79-1.74) | (0.89-6.06) | (0.73-2.49) | (0.57-2.42) |
| Poorer | 0.99 | 1.22 | 1.49 | 1.31 |  | 1.04 | 0.81 | 0.86 | 1.95 | 1.00 | 1.32 |
|  | (0.81-1.21) | (0.66-2.29) | (0.90-2.48) | (0.83-2.09) |  | (0.72-1.48) | (0.51-1.28) | (0.63-1.18) | (0.63-6.07) | (0.52-1.93) | (0.71-2.44) |
| Middle | 1.00 | 1.00 | 1.00 | 1.00 |  | 1.00 | 1.00 | 1.00 | 1.00 | 1.00 | 1.00 |
| Richer | 0.79* | 1.15 | 1.07 | 1.47 |  | 0.88 | 0.68 | 0.56*** | 3.70* | 1.23 | 1.31 |
|  | (0.63-0.99) | (0.51-2.57) | (0.67-1.73) | (0.87-2.47) |  | (0.56-1.38) | (0.44-1.06) | (0.39-0.79) | (1.11-12.25) | (0.72-2.10) | (0.70-2.45) |
| Richest | 0.79* | 0.40 | 0.91 | 1.33 |  | 0.74 | 0.75 | 0.66* | 5.60** | 1.23 | 0.67 |
|  | (0.64-0.99) | (0.15-1.04) | (0.53-1.58) | (0.62-2.85) |  | (0.45-1.22) | (0.47-1.21) | (0.48-0.90) | (1.91-16.45) | (0.69-2.19) | (0.32-1.40) |
| Marital status |  |  |  |  |  |  |  |  |  |  |  |
| Married/cohabiting | 1.00 | 1.00 | 1.00 | 1.00 |  | 1.00 | 1.00 | 1.00 | 1.00 | 1.00 | 1.00 |
| Not married | 1.12 | 1.22 | 1.00 | 0.78 |  | 1.13 | 1.00 | 1.07 | 0.77 | 1.24 | 1.24 |
|  | (0.95-1.32) | (0.70-2.13) | (0.73-1.38) | (0.51-1.18) |  | (0.80-1.60) | (0.72-1.38) | (0.83-1.38) | (0.39-1.54) | (0.77-1.99) | (0.80-1.91) |
| Current drinker | 1.21 | 1.22 | 0.97 | 0.84 |  | 1.06 | 0.85 | 1.15 | 1.55 | 1.56 | 1.18 |
|  | (0.97-1.51) | (0.78-1.91) | (0.71-1.34) | (0.60-1.18) |  | (0.73-1.55) | (0.61-1.18) | (0.71-1.86) | (0.68-3.54) | (0.83-2.92) | (0.63-2.19) |
| Current smoker | 0.98 | 1.07 | 0.84 | 2.16*** |  | 1.07 | 0.60* | 0.92 | 0.40* | 1.03 | 1.66* |
|  | (0.82-1.17) | (0.58-1.98) | (0.59-1.19) | (1.43-3.27) |  | (0.74-1.55) | (0.36-0.99) | (0.73-1.16) | (0.17-0.93) | (0.51-2.10) | (1.05-2.64) |
| Physical activity |  |  |  |  |  |  |  |  |  |  |  |
| High | 1.00 | 1.00 | 1.00 | 1.00 |  | 1.00 | 1.00 | 1.00 | 1.00 | 1.00 | 1.00 |
| Moderate | 1.02 | 0.87 | 0.94 | 0.85 |  | 1.26 | 1.22 | 1.20 | 1.09 | 0.62 | 0.89 |
|  | (0.86-1.20) | (0.48-1.58) | (0.61-1.45) | (0.53-1.35) |  | (0.80-1.99) | (0.79-1.90) | (0.94-1.53) | (0.38-3.18) | (0.38-1.01) | (0.43-1.82) |
| Low | 1.24* | 1.01 | 0.91 | 1.65* |  | 1.62* | 1.12 | 1.28 | 1.48 | 1.20 | 1.17 |
|  | (1.03-1.50) | (0.56-1.85) | (0.64-1.29) | (1.09-2.50) |  | (1.07-2.46) | (0.73-1.71) | (0.94-1.74) | (0.66-3.31) | (0.71-2.01) | (0.65-2.12) |
| Angina | 2.02*** | 2.78*** | 2.36*** | 1.78* |  | 1.75** | 1.92*** | 1.91*** | 1.84 | 2.49*** | 2.20** |
|  | (1.70-2.40) | (1.55-4.96) | (1.60-3.47) | (1.12-2.83) |  | (1.22-2.51) | (1.35-2.72) | (1.44-2.52) | (0.72-4.70) | (1.52-4.07) | (1.25-3.88) |
| Arthritis | 1.54*** | 2.46*** | 1.49* | 1.70* |  | 1.39* | 1.57** | 1.43** | 0.96 | 1.97** | 1.28 |
|  | (1.33-1.78) | (1.64-3.68) | (1.08-2.04) | (1.08-2.65) |  | (1.08-1.78) | (1.12-2.20) | (1.13-1.80) | (0.50-1.84) | (1.27-3.06) | (0.79-2.07) |
| Asthma | 1.68*** | 2.31** | 1.15 | 1.56* |  | 1.75 | 1.26 | 2.13*** | 0.62 | 1.09 | 1.68 |
|  | (1.28-2.22) | (1.40-3.82) | (0.73-1.80) | (1.05-2.34) |  | (0.89-3.44) | (0.73-2.20) | (1.41-3.20) | (0.24-1.63) | (0.59-2.01) | (0.99-2.86) |
| Chronic lung disease | 1.32 | 0.98 | 2.10*** | 2.21*** |  | 1.40 | 3.74*** | 0.81 | 1.41 | 1.82 | 3.47*** |
|  | (1.00-1.75) | (0.40-2.43) | (1.41-3.12) | (1.51-3.23) |  | (0.92-2.13) | (1.72-8.12) | (0.49-1.34) | (0.60-3.31) | (0.98-3.40) | (1.84-6.52) |
| Depression | 2.36*** | 2.62*** | 2.22*** | 4.03*** |  | 4.63*** | 1.75* | 1.94*** | 5.12*** | 3.15*** | 3.44* |
|  | (1.95-2.86) | (1.60-4.28) | (1.58-3.12) | (2.91-5.59) |  | (2.76-7.77) | (1.02-3.01) | (1.45-2.58) | (2.42-10.83) | (1.97-5.06) | (1.27-9.30) |
| Diabetes | 1.30* | 1.19 | 0.95 | 1.09 |  | 1.11 | 1.09 | 1.82** | 2.02 | 1.18 | 1.27 |
|  | (1.06-1.60) | (0.66-2.13) | (0.64-1.40) | (0.71-1.68) |  | (0.70-1.76) | (0.58-2.04) | (1.26-2.61) | (0.92-4.42) | (0.71-1.96) | (0.64-2.52) |
| Hypertension | 1.12 | 1.50 | 1.20 | 1.10 |  | 0.77 | 0.97 | 1.24 | 1.38 | 1.03 | 0.96 |
|  | (0.95-1.32) | (0.88-2.55) | (0.84-1.71) | (0.78-1.56) |  | (0.56-1.06) | (0.72-1.31) | (0.98-1.56) | (0.68-2.80) | (0.64-1.66) | (0.60-1.52) |
| Obesity^a^ | 1.05 | 0.68 | 1.21 | 1.18 |  | 1.08 | 1.38 | 0.67 | 0.56 | 1.11 | 0.92 |
|  | (0.86-1.27) | (0.41-1.15) | (0.84-1.73) | (0.82-1.71) |  | (0.66-1.78) | (0.90-2.13) | (0.38-1.21) | (0.28-1.14) | (0.73-1.70) | (0.59-1.41) |
| Stroke | 1.81*** | 1.72 | 1.26 | 0.57 |  | 2.11* | 2.14* | 2.35*** | 1.68 | 1.66 | 1.49 |
|  | (1.34-2.44) | (0.86-3.42) | (0.68-2.35) | (0.27-1.21) |  | (1.14-3.91) | (1.17-3.91) | (1.43-3.84) | (0.71-3.99) | (0.94-2.94) | (0.47-4.74) |

Abbreviation: COURAGE Collaborative Research on Ageing in Europe; SAGE WHO Study on global AGEing and adult health; S. Africa South Africa

Data are Odds Ratio (95% Confidence Intervals)

All models are mutually adjusted for all covariates in the model. The model using the overall sample is also adjusted for county.

^a^Obesity was based on measured weight and height.

* p<0.05, ** p<0.01, *** p<0.001
