# Supplementary material for: Chronic Conditions and Sleep Problems among Adults Aged 50 years or over in Nine Countries: A Multi-Country Study
Source: PLoS One. 2014 Dec 5;9(12):e114742. doi: 10.1371/journal.pone.0114742 (PMC4257709; doi:10.1371/journal.pone.0114742)
Supplement: Table S2 — Association between number of chronic conditions (independent variable) or other covariates and severe/extreme sleep problems. (DOCX) [file pone.0114742.s002.docx]

| **Table S2** Association between number of chronic conditions (independent variable) or other covariates and severe/extreme sleep problems | | | | | | | | | | | |
| --- | --- | --- | --- | --- | --- | --- | --- | --- | --- | --- | --- |
| (dependent variable) estimated by logistic regression with multiple variables | | | | | | | | | | | |
|  |  | COURAGE study | | |  | SAGE study | | | | | |
|  | Overall | Finland | Poland | Spain |  | China | Ghana | India | Mexico | Russia | S. Africa |
| Age (years) |  |  |  |  |  |  |  |  |  |  |  |
| 50-59 | 1.00 | 1.00 | 1.00 | 1.00 |  | 1.00 | 1.00 | 1.00 | 1.00 | 1.00 | 1.00 |
| 60-69 | 1.30** | 0.49* | 1.04 | 0.86 |  | 1.12 | 1.34 | 1.29* | 7.66** | 2.59** | 0.80 |
|  | (1.11-1.54) | (0.27-0.89) | (0.72-1.51) | (0.58-1.28) |  | (0.78-1.59) | (0.93-1.94) | (1.01-1.65) | (2.16-27.24) | (1.40-4.81) | (0.49-1.31) |
| 70-79 | 1.43*** | 0.46* | 1.35 | 1.22 |  | 1.02 | 1.63* | 1.43* | 4.23* | 2.69** | 1.26 |
|  | (1.18-1.73) | (0.22-0.97) | (0.87-2.09) | (0.80-1.84) |  | (0.70-1.50) | (1.08-2.45) | (1.05-1.94) | (1.12-16.01) | (1.45-4.97) | (0.60-2.64) |
| >80 | 2.03*** | 1.17 | 1.37 | 0.70 |  | 1.56 | 2.06** | 2.79*** | 6.15* | 3.88*** | 1.50 |
|  | (1.52-2.71) | (0.55-2.50) | (0.84-2.24) | (0.35-1.41) |  | (0.88-2.75) | (1.23-3.45) | (1.57-4.96) | (1.51-25.05) | (1.90-7.92) | (0.66-3.44) |
| Sex |  |  |  |  |  |  |  |  |  |  |  |
| Male | 1.00 | 1.00 | 1.00 | 1.00 |  | 1.00 | 1.00 | 1.00 | 1.00 | 1.00 | 1.00 |
| Female | 1.83*** | 1.27 | 1.27 | 2.23*** |  | 1.66** | 1.09 | 1.82*** | 2.02* | 2.10*** | 2.14*** |
|  | (1.57-2.13) | (0.81-1.99) | (0.92-1.75) | (1.40-3.57) |  | (1.16-2.38) | (0.77-1.53) | (1.43-2.33) | (1.03-3.95) | (1.40-3.17) | (1.37-3.35) |
| Education |  |  |  |  |  |  |  |  |  |  |  |
| >Tertiary | 1.00 | 1.00 | 1.00 | 1.00 |  | 1.00 | 1.00 | 1.00 | 1.00 | 1.00 | 1.00 |
| Secondary | 0.90 | 1.33 | 1.15 | 1.48 |  | 0.83 | 1.15 | 1.19 | 10.32** | 0.60 | 0.68 |
|  | (0.67-1.22) | (0.72-2.46) | (0.74-1.80) | (0.72-3.06) |  | (0.40-1.73) | (0.50-2.65) | (0.52-2.71) | (1.79-59.39) | (0.34-1.05) | (0.13-3.46) |
| <Primary | 1.37* | 1.23 | 1.22 | 1.79 |  | 1.21 | 1.30 | 1.94 | 5.94** | 1.22 | 1.19 |
|  | (1.02-1.86) | (0.55-2.75) | (0.76-1.95) | (0.84-3.82) |  | (0.55-2.64) | (0.51-3.29) | (0.84-4.45) | (1.56-22.63) | (0.69-2.15) | (0.26-5.46) |
| Wealth |  |  |  |  |  |  |  |  |  |  |  |
| Poorest | 1.21 | 1.47 | 1.58 | 1.34 |  | 1.08 | 1.23 | 1.18 | 2.06 | 1.42 | 1.42 |
|  | (0.97-1.52) | (0.71-3.06) | (0.97-2.57) | (0.78-2.30) |  | (0.71-1.66) | (0.80-1.90) | (0.81-1.72) | (0.92-4.61) | (0.77-2.63) | (0.65-3.07) |
| Poorer | 0.98 | 1.36 | 1.40 | 1.39 |  | 1.10 | 0.78 | 0.86 | 1.49 | 1.03 | 1.46 |
|  | (0.80-1.20) | (0.73-2.56) | (0.85-2.31) | (0.89-2.18) |  | (0.77-1.57) | (0.50-1.22) | (0.63-1.17) | (0.63-3.53) | (0.55-1.92) | (0.79-2.68) |
| Middle | 1.00 | 1.00 | 1.00 | 1.00 |  | 1.00 | 1.00 | 1.00 | 1.00 | 1.00 | 1.00 |
| Richer | 0.76* | 1.19 | 1.02 | 1.46 |  | 0.86 | 0.63* | 0.54*** | 3.12* | 1.25 | 1.28 |
|  | (0.61-0.95) | (0.58-2.45) | (0.62-1.66) | (0.89-2.39) |  | (0.55-1.34) | (0.41-0.98) | (0.38-0.77) | (1.18-8.28) | (0.72-2.14) | (0.72-2.27) |
| Richest | 0.72** | 0.40 | 0.87 | 1.21 |  | 0.70 | 0.68 | 0.61** | 4.39** | 1.09 | 0.67 |
|  | (0.57-0.91) | (0.15-1.05) | (0.50-1.52) | (0.58-2.55) |  | (0.43-1.16) | (0.41-1.10) | (0.45-0.84) | (1.63-11.81) | (0.59-2.04) | (0.33-1.38) |
| Marital status |  |  |  |  |  |  |  |  |  |  |  |
| Married/cohabiting | 1.00 | 1.00 | 1.00 | 1.00 |  | 1.00 | 1.00 | 1.00 | 1.00 | 1.00 | 1.00 |
| Not married | 1.11 | 1.18 | 1.03 | 0.84 |  | 1.08 | 0.99 | 1.07 | 1.02 | 1.32 | 1.12 |
|  | (0.94-1.31) | (0.70-1.99) | (0.76-1.39) | (0.56-1.25) |  | (0.76-1.53) | (0.72-1.35) | (0.84-1.38) | (0.44-2.36) | (0.77-2.26) | (0.72-1.75) |
| Current drinker | 1.18 | 1.21 | 0.97 | 0.84 |  | 1.06 | 0.87 | 1.11 | 1.58 | 1.46 | 1.09 |
|  | (0.95-1.46) | (0.77-1.89) | (0.72-1.32) | (0.60-1.19) |  | (0.73-1.53) | (0.63-1.19) | (0.69-1.77) | (0.75-3.29) | (0.76-2.81) | (0.58-2.03) |
| Current smoker | 1.01 | 1.11 | 0.84 | 2.26*** |  | 1.11 | 0.63 | 0.92 | 0.40* | 1.04 | 2.00** |
|  | (0.85-1.22) | (0.61-2.03) | (0.60-1.19) | (1.48-3.45) |  | (0.77-1.59) | (0.38-1.03) | (0.72-1.18) | (0.17-0.98) | (0.52-2.07) | (1.29-3.12) |
| Physical activity |  |  |  |  |  |  |  |  |  |  |  |
| High | 1.00 | 1.00 | 1.00 | 1.00 |  | 1.00 | 1.00 | 1.00 | 1.00 | 1.00 | 1.00 |
| Moderate | 1.02 | 0.86 | 0.93 | 0.82 |  | 1.24 | 1.21 | 1.19 | 0.87 | 0.61 | 0.77 |
|  | (0.86-1.21) | (0.48-1.54) | (0.62-1.40) | (0.51-1.31) |  | (0.79-1.95) | (0.78-1.87) | (0.93-1.51) | (0.31-2.43) | (0.37-1.01) | (0.37-1.61) |
| Low | 1.25* | 0.96 | 0.90 | 1.75** |  | 1.68* | 1.11 | 1.27 | 1.31 | 1.08 | 1.04 |
|  | (1.04-1.50) | (0.53-1.72) | (0.64-1.27) | (1.15-2.65) |  | (1.10-2.56) | (0.75-1.67) | (0.94-1.71) | (0.58-2.95) | (0.62-1.91) | (0.60-1.79) |
| No. of chronic conditions |  |  |  |  |  |  |  |  |  |  |  |
| 0 (reference) | 1.00 | 1.00 | 1.00 | 1.00 |  | 1.00 | 1.00 | 1.00 | 1.00 | 1.00 | 1.00 |
| 1 | 1.41* | 1.60 | 1.97* | 3.93** |  | 0.84 | 1.13 | 1.41 | 0.80 | 3.18 | 1.37 |
|  | (1.09-1.82) | (0.47-5.46) | (1.02-3.79) | (1.68-9.21) |  | (0.53-1.33) | (0.72-1.79) | (0.99-2.01) | (0.21-3.03) | (0.91-11.15) | (0.55-3.40) |
| 2 | 2.55*** | 3.03 | 2.65** | 4.94*** |  | 1.60* | 2.17** | 2.91*** | 2.00 | 5.01* | 1.79 |
|  | (1.99-3.27) | (0.88-10.47) | (1.35-5.20) | (2.01-12.17) |  | (1.00-2.55) | (1.36-3.47) | (2.09-4.07) | (0.61-6.56) | (1.30-19.26) | (0.69-4.67) |
| 3 | 3.22*** | 4.55* | 3.92*** | 9.80*** |  | 1.22 | 2.93*** | 3.27*** | 2.42 | 8.55** | 2.99* |
|  | (2.52-4.11) | (1.42-14.55) | (1.98-7.75) | (4.19-22.94) |  | (0.72-2.07) | (1.74-4.94) | (2.35-4.55) | (0.70-8.45) | (2.33-31.38) | (1.05-8.51) |
| 4+ | 7.62*** | 10.91*** | 8.50*** | 17.95*** |  | 4.53*** | 4.85*** | 7.71*** | 3.70 | 19.20*** | 7.10*** |
|  | (5.88-9.87) | (3.14-37.91) | (4.34-16.65) | (7.72-41.75) |  | (2.57-7.97) | (2.52-9.32) | (5.25-11.32) | (0.92-14.93) | (5.64-65.37) | (2.63-19.13) |

Abbreviation: COURAGE Collaborative Research on Ageing in Europe; SAGE WHO Study on global AGEing and adult health; S. Africa South Africa

Data are Odds Ratio (95% Confidence Intervals)

All models are mutually adjusted for all covariates in the model. The model using the overall sample is also adjusted for county.

* p<0.05, ** p<0.01, *** p<0.001
