# Supplementary material for: Chronic Conditions and Sleep Problems among Adults Aged 50 years or over in Nine Countries: A Multi-Country Study
Source: PLoS One. 2014 Dec 5;9(12):e114742. doi: 10.1371/journal.pone.0114742 (PMC4257709; doi:10.1371/journal.pone.0114742)
Supplement: Table S3 — Prevalence of severe/extreme sleep problems by presence of chronic condition and country among adults aged 50 years or over (self-reported diagnosis). (DOCX) [file pone.0114742.s003.docx]

**Table S3** Prevalence of severe/extreme sleep problems by presence of chronic condition and country among adults aged 50 years or over (self-reported diagnosis)

|  |  |  |  | COURAGE study | | | | |  |  |  |  | SAGE study | | | |  |  |  |  |  |  |  |  |  |  |  |  |  |
| --- | --- | --- | --- | --- | --- | --- | --- | --- | --- | --- | --- | --- | --- | --- | --- | --- | --- | --- | --- | --- | --- | --- | --- | --- | --- | --- | --- | --- | --- |
|  | Overall | |  | Finland | |  | Poland | |  | Spain |  |  | China | |  | Ghana | |  | India |  |  | Mexico | |  | Russia | |  | S. Africa | |
| Chronic condition | Yes | No |  | Yes | No |  | Yes | No |  | Yes | No |  | Yes | No |  | Yes | No |  | Yes | No |  | Yes | No |  | Yes | No |  | Yes | No |
| Angina^a,b,c,d,e,f,g,i,j^ | 17.3 | 8.2 |  | 20.6 | 8.2 |  | 35.6 | 14.9 |  | 17.6 | 8.4 |  | 5.7 | 2.5 |  | 19.6 | 7.0 |  | 27.5 | 13.8 |  | 9.6 | 5.5 |  | 17.0 | 6.2 |  | 15.5 | 9.1 |
|  | (1.4) | (0.4) |  | (3.5) | (0.8) |  | (3.4) | (1.0) |  | (2.8) | (0.8) |  | (0.8) | (0.2) |  | (4.0) | (0.5) |  | (3.0) | (0.9) |  | (6.1) | (1.3) |  | (2.5) | (1.0) |  | (3.6) | (0.7) |
| Arthritis^a,b,c,d,e,f,g,i,j^ | 15.1 | 7.5 |  | 15.0 | 5.2 |  | 27.2 | 13.5 |  | 16.7 | 5.8 |  | 4.4 | 2.3 |  | 10.2 | 6.9 |  | 22.2 | 12.8 |  | 8.7 | 5.4 |  | 17.8 | 6.2 |  | 14.6 | 7.7 |
|  | (0.9) | (0.4) |  | (1.2) | (0.7) |  | (2.0) | (1.0) |  | (1.6) | (0.7) |  | (0.5) | (0.2) |  | (1.5) | (0.6) |  | (2.2) | (0.8) |  | (2.5) | (1.4) |  | (2.8) | (0.8) |  | (1.9) | (0.7) |
| Asthma^a,b,c,d,g,j^ | 20.2 | 8.8 |  | 21.8 | 8.2 |  | 31.4 | 16.9 |  | 18.4 | 8.2 |  | 4.8 | 2.7 |  | 8.2 | 7.4 |  | 25.0 | 13.7 |  | 5.1 | 5.7 |  | 12.2 | 9.6 |  | 21.4 | 8.8 |
|  | (2.6) | (0.4) |  | (3.2) | (0.6) |  | (4.3) | (1.0) |  | (2.4) | (0.8) |  | (1.7) | (0.2) |  | (2.2) | (0.6) |  | (5.0) | (0.8) |  | (2.8) | (1.3) |  | (4.0) | (1.1) |  | (5.3) | (0.7) |
| Chronic lung | 16.4 | 8.7 |  | 15.0 | 9.4 |  | 34.1 | 16.3 |  | 23.3 | 7.7 |  | 4.9 | 2.6 |  | 15.9 | 7.4 |  | 25.1 | 14.0 |  | 12.0 | 5.4 |  | 18.6 | 8.2 |  | 22.9 | 9.0 |
| disease^a,c,d,e,g,h,i,j^ | (1.4) | (0.4) |  | (4.4) | (0.7) |  | (4.1) | (1.0) |  | (2.9) | (0.7) |  | (0.9) | (0.2) |  | (7.3) | (0.5) |  | (3.9) | (0.8) |  | (3.4) | (1.3) |  | (2.6) | (1.1) |  | (7.2) | (0.7) |
| Depression^a,b,c,d,e,f,g,i,j^ | 24.5 | 8.6 |  | 22.1 | 7.5 |  | 34.3 | 15.8 |  | 18.7 | 6.2 |  | 9.3 | 2.7 |  | 19.1 | 7.2 |  | 26.9 | 14.0 |  | 10.3 | 4.9 |  | 27.7 | 9.0 |  | 22.5 | 9.0 |
|  | (1.7) | (0.4) |  | (3.3) | (0.7) |  | (3.7) | (1.0) |  | (2.1) | (0.6) |  | (5.6) | (0.2) |  | (6.1) | (0.5) |  | (3.9) | (0.8) |  | (5.4) | (1.2) |  | (4.7) | (1.1) |  | (6.3) | (0.7) |
| Hypertension^a,b,c,d,f,g,h,i^ | 12.4 | 7.9 |  | 13.9 | 6.6 |  | 21.9 | 13.2 |  | 11.9 | 6.9 |  | 3.1 | 2.6 |  | 10.1 | 7.0 |  | 22.7 | 12.9 |  | 11.3 | 3.2 |  | 12.5 | 6.5 |  | 11.1 | 8.7 |
|  | (0.7) | (0.4) |  | (1.4) | (0.7) |  | (1.4) | (1.2) |  | (1.4) | (0.6) |  | (0.4) | (0.2) |  | (1.5) | (0.5) |  | (2.0) | (0.9) |  | (3.4) | (0.7) |  | (1.7) | (1.1) |  | (1.6) | (0.8) |
| Stroke^a,b,c,e,f,g,i^ | 19.3 | 8.9 |  | 17.6 | 9.2 |  | 27.5 | 17.3 |  | 10.5 | 8.9 |  | 5.3 | 2.7 |  | 21.6 | 7.0 |  | 35.0 | 14.1 |  | 11.7 | 5.4 |  | 26.1 | 8.9 |  | 14.8 | 9.2 |
|  | (2.2) | (0.4) |  | (4.4) | (0.7) |  | (5.2) | (1.0) |  | (4.4) | (0.8) |  | (1.3) | (0.2) |  | (4.5) | (0.5) |  | (7.0) | (0.9) |  | (4.0) | (1.3) |  | (5.7) | (1.1) |  | (5.2) | (0.7) |

Abbreviation: COURAGE Collaborative Research on Ageing in Europe; SAGE WHO Study on global AGEing and adult health; S. Africa South Africa

Data are % (SE). % is the percentage of individuals with sleep problems by the presence (Yes) or absence (No) of that chronic condition.

Results for diabetes and obesity are not shown as they are the same as the results in table 2 in the text as there were no symptom-based algorithms for diabetes and obesity was based only on measured weight and height.

Difference between individuals with and without that chronic condition is statistically significant (P<0.05) in ^a^Overall sample, ^b^Finland, ^c^Poland, ^d^Spain, ^e^China, ^f^Ghana, ^g^India, ^h^Mexico, ^i^Russia, and ^j^South Africa.
