# Supplementary material for: Chronic Conditions and Sleep Problems among Adults Aged 50 years or over in Nine Countries: A Multi-Country Study
Source: PLoS One. 2014 Dec 5;9(12):e114742. doi: 10.1371/journal.pone.0114742 (PMC4257709; doi:10.1371/journal.pone.0114742)
Supplement: Table S4 — Association between chronic conditions (independent variable) and severe/extreme sleep problems (dependent variable) among adults aged 50 years or over estimated by logistic regression with multiple variables (self-reported diagnosis). (DOCX) [file pone.0114742.s004.docx]

| **Table S4** Association between chronic conditions (independent variable) and severe/extreme sleep problems (dependent variable) among adults aged 50 years or over estimated by logistic regression with multiple variables (self-reported diagnosis) | | | | | | | | | | | |
| --- | --- | --- | --- | --- | --- | --- | --- | --- | --- | --- | --- |
|  |  | COURAGE study | |  |  | SAGE study | |  |  |  |  |
|  | Overall | Finland | Poland | Spain |  | China | Ghana | India | Mexico | Russia | S. Africa |
| Angina | 1.96*** | 2.04* | 1.99*** | 1.93* |  | 1.83** | 2.62*** | 1.92** | 0.88 | 2.27*** | 1.68 |
|  | (1.58-2.43) | (1.02-4.09) | (1.33-2.99) | (1.16-3.21) |  | (1.23-2.71) | (1.49-4.58) | (1.20-3.08) | (0.15-5.25) | (1.45-3.53) | (0.90-3.12) |
| Arthritis | 1.54*** | 2.75*** | 1.57** | 1.58* |  | 1.60*** | 1.11 | 1.37* | 1.55 | 2.02** | 1.51 |
|  | (1.32-1.80) | (1.94-3.91) | (1.18-2.09) | (1.00-2.49) |  | (1.25-2.04) | (0.73-1.68) | (1.05-1.79) | (0.77-3.14) | (1.33-3.08) | (0.94-2.41) |
| Asthma | 1.45 | 2.60*** | 1.07 | 1.46 |  | 1.02 | 0.88 | 1.69 | 0.37 | 0.77 | 2.87** |
|  | (0.97-2.14) | (1.57-4.31) | (0.62-1.84) | (0.90-2.37) |  | (0.40-2.58) | (0.45-1.71) | (0.91-3.13) | (0.10-1.46) | (0.29-2.04) | (1.40-5.87) |
| Chronic lung disease | 1.78*** | 0.84 | 2.10** | 2.63*** |  | 1.68* | 2.21 | 1.30 | 1.76 | 2.18** | 2.46* |
|  | (1.37-2.31) | (0.30-2.39) | (1.32-3.34) | (1.67-4.15) |  | (1.11-2.52) | (0.56-8.69) | (0.76-2.20) | (0.83-3.71) | (1.22-3.92) | (1.12-5.43) |
| Depression | 2.10*** | 2.87*** | 2.16*** | 2.74*** |  | 1.20 | 2.58* | 1.60* | 2.45* | 2.72*** | 1.83 |
|  | (1.70-2.59) | (1.73-4.75) | (1.46-3.19) | (2.02-3.73) |  | (0.16-9.09) | (1.17-5.68) | (1.06-2.41) | (1.02-5.90) | (1.59-4.65) | (0.77-4.31) |
| Diabetes | 1.28* | 1.05 | 0.94 | 1.14 |  | 1.14 | 1.08 | 1.66** | 1.96 | 1.24 | 1.35 |
|  | (1.04-1.58) | (0.59-1.86) | (0.65-1.38) | (0.74-1.74) |  | (0.71-1.84) | (0.61-1.93) | (1.14-2.42) | (0.93-4.13) | (0.74-2.10) | (0.64-2.82) |
| Hypertension | 1.34** | 2.49*** | 1.36 | 1.25 |  | 0.87 | 1.18 | 1.75*** | 2.38** | 0.97 | 1.19 |
|  | (1.13-1.58) | (1.52-4.08) | (1.00-1.86) | (0.88-1.79) |  | (0.65-1.16) | (0.79-1.75) | (1.32-2.31) | (1.38-4.12) | (0.62-1.51) | (0.72-1.96) |
| Obesity^a^ | 1.04 | 0.63 | 1.20 | 1.25 |  | 1.06 | 1.30 | 0.71 | 0.52 | 1.12 | 0.83 |
|  | (0.85-1.28) | (0.38-1.03) | (0.84-1.72) | (0.88-1.78) |  | (0.67-1.68) | (0.85-1.99) | (0.39-1.30) | (0.24-1.10) | (0.71-1.74) | (0.55-1.25) |
| Stroke | 1.95*** | 1.44 | 1.50 | 0.43 |  | 1.80* | 2.25* | 2.88** | 1.67 | 2.18* | 1.43 |
|  | (1.40-2.72) | (0.69-3.00) | (0.78-2.89) | (0.17-1.06) |  | (1.05-3.10) | (1.15-4.42) | (1.51-5.49) | (0.61-4.56) | (1.20-3.97) | (0.41-5.00) |

Abbreviation: COURAGE Collaborative Research on Ageing in Europe; SAGE WHO Study on global AGEing and adult health; S. Africa South Africa

Data are Odds Ratio (95% Confidence Intervals)

All models are mutually adjusted for all chronic conditions in the model and age, sex, education, wealth, marital status, alcohol consumption, smoking, and physical activity. The model using the overall sample is also adjusted for county.

^a^Obesity was based on measured weight and height.

* p<0.05, ** p<0.01, *** p<0.001
