# Supplementary material for: Chronic Conditions and Sleep Problems among Adults Aged 50 years or over in Nine Countries: A Multi-Country Study
Source: PLoS One. 2014 Dec 5;9(12):e114742. doi: 10.1371/journal.pone.0114742 (PMC4257709; doi:10.1371/journal.pone.0114742)
Supplement: Table S5 — Association between number of chronic conditions (independent variable) and severe/extreme sleep problems (dependent variable) estimated by logistic regression with multiple variables (self-reported diagnosis). (DOCX) [file pone.0114742.s005.docx]

| **Table S5** Association between number of chronic conditions (independent variable) and severe/extreme sleep problems (dependent variable) estimated by logistic regression with multiple variables (self-reported diagnosis) | | | | | | | | | | | |
| --- | --- | --- | --- | --- | --- | --- | --- | --- | --- | --- | --- |
| No. of chronic |  | COURAGE study | |  |  | SAGE study | |  |  |  |  |
| conditions^a^ | Overall | Finland | Poland | Spain |  | China | Ghana | India | Mexico | Russia | S. Africa |
| 0 (reference) | 1.00 | 1.00 | 1.00 | 1.00 |  | 1.00 | 1.00 | 1.00 | 1.00 | 1.00 | 1.00 |
| 1 | 1.64*** | 2.70 | 1.70 | 1.47 |  | 1.11 | 1.93*** | 1.84*** | 0.43 | 2.02 | 1.19 |
|  | (1.36-1.99) | (0.84-8.65) | (0.97-2.97) | (0.76-2.85) |  | (0.81-1.53) | (1.38-2.71) | (1.41-2.39) | (0.16-1.11) | (0.78-5.20) | (0.71-2.01) |
| 2 | 2.26*** | 5.39** | 2.42** | 2.69** |  | 1.45 | 2.01** | 2.43*** | 3.15** | 3.27* | 1.84* |
|  | (1.79-2.85) | (1.82-15.98) | (1.41-4.14) | (1.45-4.97) |  | (0.97-2.17) | (1.25-3.23) | (1.67-3.54) | (1.33-7.45) | (1.30-8.20) | (1.06-3.18) |
| 3 | 3.38*** | 8.67*** | 3.27*** | 5.03*** |  | 1.87* | 1.25 | 4.02*** | 1.25 | 4.53*** | 2.11* |
|  | (2.62-4.37) | (2.92-25.71) | (1.89-5.65) | (2.71-9.35) |  | (1.01-3.49) | (0.54-2.90) | (2.22-7.27) | (0.44-3.53) | (2.18-9.39) | (1.12-3.97) |
| 4+ | 6.70*** | 17.52*** | 6.79*** | 6.87*** |  | 4.54*** | 7.56*** | 7.59*** | 3.34* | 9.65*** | 4.56*** |
|  | (5.09-8.82) | (5.51-55.75) | (3.82-12.09) | (3.47-13.59) |  | (2.45-8.42) | (2.74-20.85) | (3.93-14.67) | (1.06-10.54) | (4.48-20.79) | (2.11-9.87) |

Abbreviation: COURAGE Collaborative Research on Ageing in Europe; SAGE WHO Study on global AGEing and adult health; S. Africa South Africa

Data are Odds Ratio (95% Confidence Intervals)

Trend test was significant for all regression analyses (p<0.001).

^a^ Number of chronic conditions was based on self-reported diagnosis with the exception of obesity which was based on measured weight and height.

All models are mutually adjusted for age, sex, education, wealth, marital status, alcohol consumption, smoking, and physical activity. The model using the overall sample is also adjusted for county.

* p<0.05, ** p<0.01, *** p<0.001
